# Hydrodynamic Modelling and Flood Risk Analysis of Urban Catchments under Multiple Scenarios: A Case Study of Dongfeng Canal District, Zhengzhou

**DOI:** 10.3390/ijerph192214630

**Published:** 2022-11-08

**Authors:** Huaibin Wei, Liyuan Zhang, Jing Liu

**Affiliations:** 1 School of Management and Economics, North China University of Water Resources and Electric Power, Zhengzhou 450046, China; 2 School of Water Conservancy, North China University of Water Resources and Electric Power, Zhengzhou 450046, China; 3 College of Water Resources, North China University of Water Resources and Electric Power, Zhengzhou 450046, China; 4 Henan Key Laboratory of Water Resources Conservation and Intensive Utilization in the Yellow River Basin, Zhengzhou 450046, China

**Keywords:** urban flood simulation, risk assessment, InfoWorks ICM, urban drainage 1D/2D modelling

## Abstract

In recent years, urban flooding has become an increasingly serious problem, posing a serious threat to socio-economic development and personal safety. In this paper, we consider the Dongfeng Canal area in Zhengzhou City as an example and build a 1D/2D coupled urban flood model using the InfoWorks ICM. This study area uses six scenarios with rainfall return periods of 5 a, 20 a, and 50 a, corresponding to rainfall ephemeris of 1 h and 2 h to assess the flood risk. The results of the study show that (1) The flood depth, inundation duration, and extent of inundation in the study area vary with the return period and rainfall history. Generally, most of the water accumulation is concentrated in the low-lying areas adjacent to the river and near the roadbed. (2) As the rainfall recurrence period and rainfall duration increase, the proportion of overflow at the nodes becomes more pronounced and the overload from the pipe network flows mainly to the overload. (3) The high-risk areas under the different scenarios are mainly distributed on both sides of the river, and most of the low-risk areas transform into medium- and high-risk areas as the rainfall recurrence period and rainfall duration increase. This study analyses the flood risk situation under different scenarios, as well as the elements and areas that should be monitored in case of flooding, with the aim of providing a reference for flood prevention and control in the study area and formulating corresponding countermeasures. It also serves as a reference for flood risk analysis in other areas with similar situations.

## 1. Introduction

Urbanisation and changes in land use have had a considerable impact on the processes and elements of the water cycle [1,2], whereas in recent years, global climate change and extreme weather have occurred frequently, and urban flooding caused by heavy rainfall has gradually become a hot topic of concern for scholars [3]. The frequent occurrence of urban flooding disasters has brought substantial economic losses and casualties to society and seriously threatened urban public safety [4]. Therefore, it is essential to understand the risk areas of urban flooding and conduct flood risk assessments to prevent and control urban flooding and reduce the losses caused by such disasters [5].

A common method for conducting an urban flood risk assessment is the historical disaster statistics method [6], which focuses on using statistical methods to analyse the development pattern of flooding, predict possible future flooding hazards, and estimate possible losses due to flooding based on historical flooding information and rainfall data in the study area [7]. For example, Hans de Moel et al. analysed trends in flood risk in time and space and made projections for future land use and flood inundation risk in the Netherlands. Their findings show that over spatial spans, flood losses are greater in areas of high economic growth than in areas of low economic growth. However, high-economic-growth areas are more resilient to flood risk than low-economic-growth areas [2]. Man Qi et al. analysed the effects of topography, rainfall, and impervious surfaces on urban flooding and their spatial patterns of variation for four recent storm events in Cincinnati, USA. They used the kriging interpolation of estimated rainfall depths to measure the impact of rainfall on urban flood hazards [8].

With the development of computer technologies, hydrological hydrodynamic simulation methods are increasingly being applied to urban flood risk assessment. Some of the more widely used numerical models in urban hydrological simulations are SWMM, MIKE, and InfoWorks ICM [9]. For example, Zhao et al. used the coupling of two models, SWMM and MIKE21, to simulate in detail the spatial distribution and water depth of the inundated area in the central part of Cangzhou City under different rainfall return periods and evaluate the economic losses from flooding in the risk area [10]. Sidek et al. used the InfoWorks ICM hydrological-hydraulic model of the Baisala basin as an example to model the response of the basin to rainfall based on the Probabilistic Distributed Moisture (PDM) model to generate flood hazard maps based on several average repetition intervals (ARI) and uniform rainfall depths and analyse the main influences affecting the flood depth and extent [11]. Tabari et al. used the InfoWorks ICM hydraulic model to quantify the impact of anthropogenic climate on urban rainfall flooding in Antwerp, Belgium, using a risk assessment framework and causal counterfactual probability theory [12]. Compared to the analysis of flood risk through historical hazard scenarios, hydraulic modelling provides a more accurate risk assessment method and allows for a more comprehensive analysis of flood risk conditions [13,14,15,16,17]. The existing models work well on a large regional scale. For urban areas, flood risk modelling needs to be more precise, for example, down to a particular road or square. However, the lack of monitoring of relevant data at the urban scale, such as check-well level data and data from drainage networks, has led to only a few academics working on flood risk models for small urban catchments [16,18,19,20,21,22,23,24,25,26], so this paper constructs an urban flooding model using the Dongfeng canal area in Zhengzhou City as an example to analyse the flood risk within this small urban catchment.

The objectives of this study are (1) to comprehensively consider the hydrological processes between river–urban drainage system–surface runoff and construct a 1D/2D coupled urban flood model based on InfoWorks ICM and an analysis of urban flood processes; (2) to use the storm intensity formulae to design different rainfall scenarios; analyse the inundation depth, duration of inundation, and inundation extent under different recurrence period design storms; and analyse the overflow distribution and drainage capacity of the pipe network at the nodes; and (3) to construct an urban flood risk assessment system to analyse the urban flood risk for the study area.

## 2. Materials and Methods

### 2.1. Study Area

Zhengzhou is the capital of Henan Province, located in the north-central part of Henan Province, where the middle and lower reaches of the Yellow River divide, between longitude $112^{\circ }42^{\prime }$–$114^{\circ }14^{\prime }$ E and latitude $34^{\circ }16^{\prime }$–$34^{\circ }58^{\prime }$ N. Zhengzhou is mostly a plain, except for the hills in the southwest, and the terrain is flat, with elevations generally less than 284 m, the lowest being only 79 m. There is a difference of 205 m between the highest and lowest points in the territory.

The Dongfeng Canal drainage area of Zhengzhou City was selected as the study area for this study. This study area is located in the northern part of the main urban area of Zhengzhou. The study area covers an area of approximately 80 km^2^.

The study area has a temperate continental monsoon climate with an average annual precipitation of 632.4 mm and an average of 78 days of precipitation per year [27]. The extreme annual maximum rainfall is 1339 mm and the extreme annual minimum rainfall is 380.6 mm, with rainfall concentrated between June and August each year and the heaviest rainfall occurring in August.

**Figure 1:**
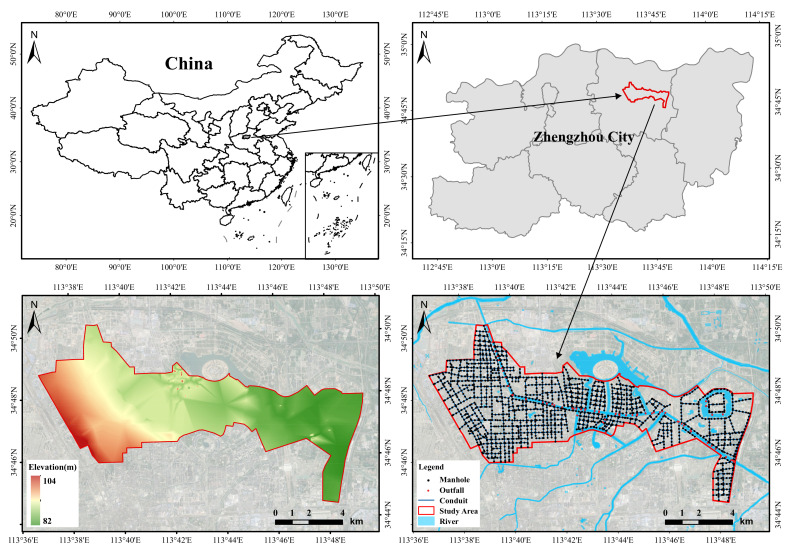
Location of the study area, stormwater network distribution, and elevation data.

The main major river network in the study area is the Dongfeng Canal. The Dongfeng Canal is a man-made river that was dug in 1958. Originally used as a channel to divert water from the Yellow River for irrigation, it now fulfils important functions in flood control, ecology, and landscape. The Dongfeng Canal starts at the Yellow River embankment and joins the Sosu and Jalu rivers to the south. At the same time, the Dongfeng Canal also intersects with a number of tributaries in the city centre. The Dongfeng Canal is one of the most important north–south oriented rivers in the city and has the task of draining flood water. The total length of the stormwater pipe network laid in the study area is 295.62 km. Stormwater in the study area is mainly discharged into the Dongfeng Drainage Canal through the stormwater pipe network laid on arterial roads such as the North Third Ring Road, Garden Road, Zhongzhou Avenue, East Yellow River Road, and East Dongfeng Road. Figure 1 shows the location of the study area, the distribution of the stormwater pipe network, and the elevation schematic.

### 2.2. Data Collection and Manipulation

The basic data required to build the hydrodynamic model during the study included the following: road-building data, stormwater pipe network and node distribution data, 5 m accuracy DEM data, and land use/land cover data.

Road construction data were obtained from OSM (https://www.openstreetmap.org (accessed on 6 April 2022)). Stormwater pipe network and node data with a 5 m accuracy provided by Zhengzhou Planning and Survey Design Institute were used for the construction of the 1D stormwater pipe network and 2D surface diffuse flow model underlying the study area.

Land use data were classified using ArcGIS PRO for the supervised classification of satellite image data. Image data were obtained from the Geospatial Data Cloud (www.gscloud.cn (accessed on 6 April 2022)), Landsat8 OLI-TIRS remote sensing imagery [28]. Based on the current land use situation in the study area and the needs of the study, the land use in the study area was classified into five categories: building land, green space, water bodies, bare soil, and roads.

The river network data in the study were river shapes determined using satellite imagery and DEM data were used to extract features from river cross-sections.

### 2.3. Research Methodology

#### 2.3.1. InfoWorks ICM Hydrodynamic Modelling

(1) Basic theory

This study integrates the water exchange between the pipe network and the two-dimensional surface and river channels and uses InfoWorks ICM software to construct a one-two-dimensional coupled urban flood model [29].

The model is mainly concerned with hydrohydraulic processes such as rainfall, surface runoff, and the drainage of the pipe network [30]. The Infoworks ICM model is used to simulate the diffusion and transport of water in pipes by completely solving the system of St.Venant equations with the control equations in Equations (1) and (2).

(1) $$\frac{\partial A}{\partial t}+\frac{\partial Q}{\partial x}=0$$

(2) $$\frac{\partial A}{\partial t}+\frac{\partial }{\partial x}\left(\frac{Q^{2}}{A}\right)+gA\left(\cos\theta \frac{\partial h}{\partial x}-S_{0}+\frac{Q\left|Q\right|}{K^{2}}\right)=0$$

where *Q* is the flow rate, m^3^/s; *A* is the pipe section area, m^2^; *t* is the time, s; *x* is the length of the pipe along the flow direction, m; *h* is the water depth, m; *g* is the acceleration of gravity, m/s; θ is the horizontal angle in degrees; *K* is the water transfer rate, determined by Manning’s formula; *S*_0_ is the slope of the pipe bottom.

The Infoworks ICM model generalises the river channel to a piped open channel when simulating the flood evolution of the river network and uses a one-dimensional hydrodynamic model for the simulation [31,32], with the basic control equations being

(3) $$\frac{\partial A}{\partial t}+\frac{\partial Q}{\partial x}=q$$

(4) $$\frac{\partial A}{\partial t}+\frac{\partial }{\partial x}\beta \left(\frac{Q^{2}}{A}\right)+gA\left(\frac{\partial y}{\partial x}\right)+gAS_{f}-uq=0$$

where *Q* is the flow rate, m^3^/s; *A* is the cross-sectional area of the river crossing, m^2^; *t* is the time, s; *x* is the horizontal coordinate along the flow direction, m; *y* is the water level, m; *g* is the acceleration of gravity; β is the momentum correction factor in degrees; *K* is the water transfer rate, determined by Manning’s formula; $S_{f}$ is the frictional slope drop; *u* is the flow rate of the lateral incoming flow in the river direction; *q* is the lateral incoming flow rate of the river, m^3^/s.

It uses the two-dimensional finite volume method to solve the shallow water equations in the simulation of two-dimensional surface diffuse flow by using the TVD excitation technique and the Riemann solver to solve the model computationally. The two-dimensional surface model can effectively and accurately simulate the flow of water on complex urban surfaces and provide support for engineering planning and design [31,33]. The shallow water control equations used in the simulation are as follows:

(5) $$\frac{\partial h}{\partial t}+\frac{\partial \left(hu\right)}{\partial x}+\frac{\partial \left(hv\right)}{\partial y}=q_{1D}$$

(6) $$\frac{\partial \left(hu\right)}{\partial t}+\frac{\partial }{\partial x}\left(hu^{2}+\frac{gh^{2}}{2}\right)+\frac{\partial \left(huv\right)}{\partial y}=S_{0,x}-S_{f,x}+q_{1D}u_{1D}$$

(7) $$\frac{\partial \left(hv\right)}{\partial t}+\frac{\partial }{\partial x}\left(hv^{2}+\frac{gh^{2}}{2}\right)+\frac{\partial \left(huv\right)}{\partial y}=S_{0,y}-S_{f,y}+q_{1D}v_{1D}$$

where *h* is the water depth, m; *u* is the velocity component in the x-direction, m/s; *v* is the velocity component in the y-direction; $S_{0,x}$ is the bottom slope component in the x-direction; $S_{0,y}$ is the bottom slope component in the y-direction; $S_{f,x}$ is the friction component in the x-direction; $S_{f,y}$ is the friction component in the y-direction; $q_{1D}$ is the outflow rate per unit area, m3/s; u_1D_ is the velocity component of $q_{1D}$ in the x-direction, m/s; $v_{1D}$ is the velocity component of $q_{1D}$ in the y-direction, m/s.

(2) One-dimensional stormwater pipe network data pre-processing

Before constructing a 1D drainage model of the study area, the raw data were pre-processed, for example, by checking the connections to the pipe network. For areas without a drainage network, the discharge of rainwater directly into the nearby mains network was considered. To reduce the calculated pressure, the stormwater pipe network was generalised. For example, pipes with several branches located in the same catchment area were combined into one drainage pipe based on their drainage capacity. After the simplification, the total length of the stormwater pipes was 295.62 km, with a total of 1837 inspection wells and 69 outlets.

(3) Catchment delineation

The overall topographical variation in the study area was not significant and the data from the stormwater network were relatively similar, so the Tyson polygon method was used for sub-catchment delineation. The Tyson polygon method was used to delineate the sub-catchments. All adjacent inspection shafts were joined into a triangle and the perpendicular bisectors of the sides of these triangles were made so that a number of perpendicular bisectors around each inspection shaft formed a polygon. In addition, some sub-basins were manually adjusted according to the layout of the pipe network and field surveys. A total of 1837 sub-catchments were finally delineated.

(4) Determination of model parameters

The flow-producing surfaces were divided into four categories according to land use: building sites, roads, green spaces, and bare soil. Green areas and bare soil are permeable surfaces. The fixed runoff coefficient method was used for impervious surfaces and the Horton method was used for permeable surfaces [32]. The SWMM nonlinear reservoir method was used to simulate the confluence of the study area based on topographic data. The parameters of different types of flow-producing surfaces were also set according to previous research results in similar areas [34,35,36,37]. The specific values of the parameters are given in Table 1.

(5) Two-dimensional model setup

The gridding interval was set for the study area and the size of the triangular grid was adjusted to meet the study requirements. Considering that the accuracy of the DEM may not reflect the inundation of the road, the grid elevation of the area where the road is located was reduced by 15 cm in order to better simulate the actual road conditions in the study area. In order to be able to reduce the amount of computation as much as possible while meeting the conditions of simulation accuracy, in the modelling process, the calculation grid was encrypted for key areas such as roads, and as large a grid as possible was used for areas with a single land use type. The final triangular grid was divided into 97,112, with a minimum grid area of 20 m^2^ and a maximum grid area of 1000 m^2^.

(6) Validation of the model

In order to check the applicability of the parameters in the study area, the simulation results need to be validated. A rainfall event with a 50-year rainfall intensity was used as the boundary condition for the model to compare the distribution of the simulated flooding points in the study area with the actual flooding points. The actual distribution of the flooding points was obtained from the information on flood-prone points published by the Zhengzhou traffic department.

**Figure 2:**
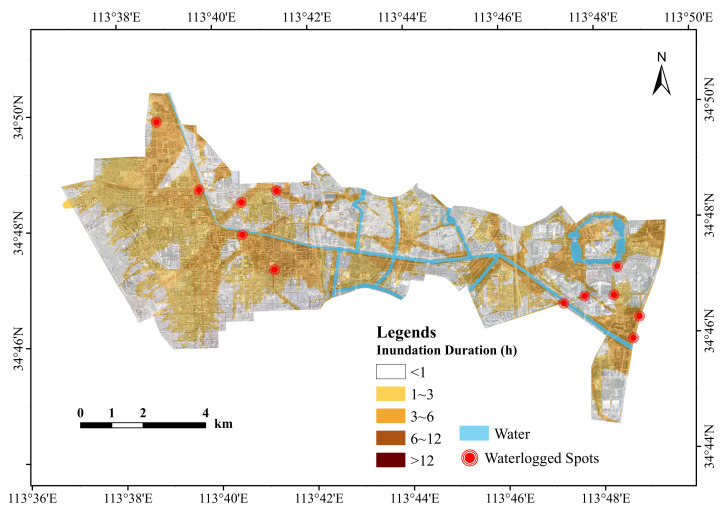
Distribution of simulated results compared to measured water accumulation points.

Comparing the historical statistical inundation points in red in Figure 2 with the inundation extent of the simulation results, it can be seen from the figure that the inundation points simulated by the model match the distribution of the actual inundation points. Most of these inundation points are located where the duration of inundation is relatively long. The model parameters are therefore considered to be appropriate and the simulation results are reliable.

#### 2.3.2. Scenario Setting

In this study area, several sets of rainfall scenarios were set up based on the storm intensity formula to analyse the flooding impacts of different rainfall scenarios [37]. The storm intensity equations used for the study are as follows:

(8) $$q=\frac{7057.6\left(1+0.794{\mathrm{lg}}_{}P\right)}{{\left(t+25.8\right)}^{0.948}}$$

where *q* is the average storm intensity in mm/min; *P* is the return period in a; *t* is the rainfall time in min.

**Figure 3:**
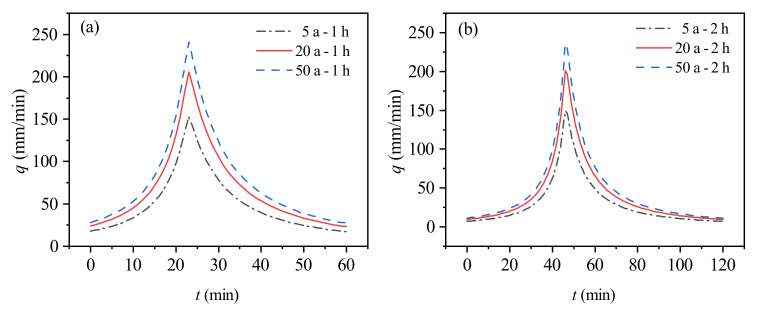
Rainfall processes with different rainfall return periods and different rainfall durations: (**a**) Rainfall duration of 1 h rainfall process. (**b**) Rainfall duration of 2 h rainfall process.

The design recurrence period of the pipe network in the study area is 2 a. Combining the historical rainfall and flood control needs of the study area, the flooding situation of the Dongfeng canal area was simulated under the rainstorm scenario, and six rainstorm scenarios with a rainfall duration of 1 h and 2 h for 5 a, 20 a, and 50 a events were designed as the different rainfall scenario driving models. The rainfall process time interval was set to 5 min and the design rainfall process line was obtained according to the rainfall intensity formula and using the ICM design rainfall generator as shown in Figure 3.

#### 2.3.3. Flood Risk Analysis Methodology

According to the UK Environmental Protection Agency, the flood risk rate is calculated by combining two key physical variables, water depth and flow velocity. During the calculation of flood risk rates, the type of subsurface is also considered to be an important factor [38]. The calculation is as follows:

(9) $$R_{H}=h\left(v+0.5\right)+\mathrm{CDF}$$

where $R_{H}$ is the risk rate, with a scale of one; *h* is the flood inundation depth, m; *v* is the flood flow velocity, m/s; CDF is the debris factor, i.e., the increased risk factor due to debris carried in the flood.

The CDF is mainly used to increase the weight of the impact of road floats on flood risk and is often used in flood risk analysis [39,40]. The CDF is assigned to 1 if the type of bedding surface is a road or a building site and the flood velocity is greater than 2, whereas the rest are assigned to 0. The flood risk rate value $R_{H}$ was quantified for any point in the study area. In addition, the flood risk rating was divided into four zones. When $R_{H}$ is less than 0.75, it means that the area is in a low-risk zone; when Rh is between 0.75 and 1.25, it means a medium-risk zone; when Rh is between 1.25 and 2.00, it means a high-risk zone; and when Rh is greater than 2.00, it means that the current area is in a very-high-risk zone. The flood risk classification is shown in Table 2.

**Figure 4:**
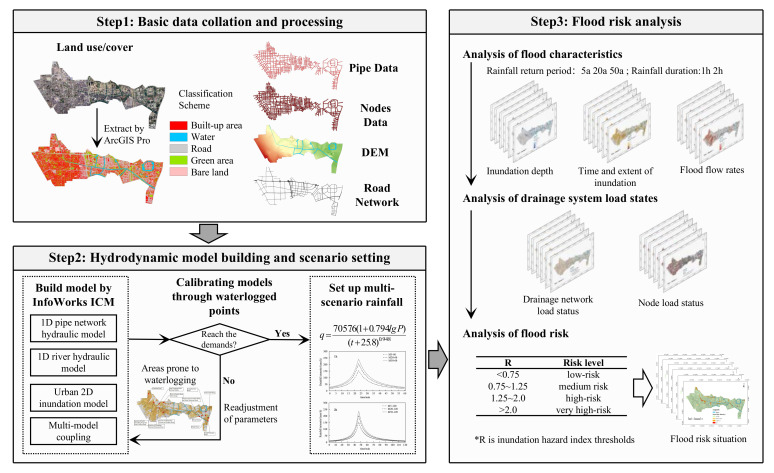
Distribution of simulated results compared to measured water accumulation points.

The methodological route of the study is shown in Figure 4.

## 3. Results and Discussion

### 3.1. Analysis of Urban Flood Simulation Results

#### 3.1.1. Analysis of Flood Inundation Water Depth

**Figure 5:**
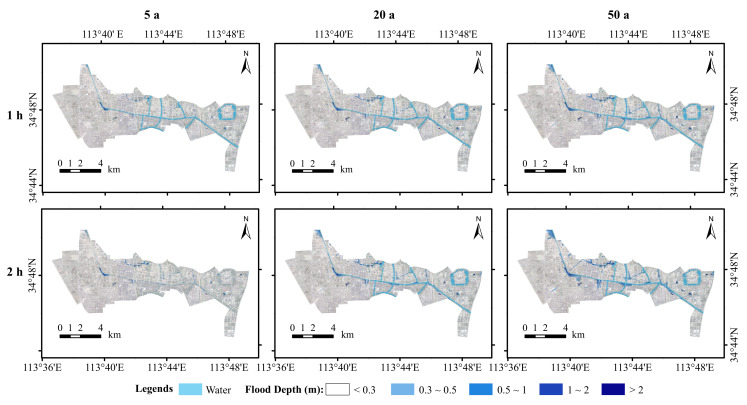
Simulated inundation depth map.

As a result of the InfoWorks ICM simulations conducted in the study area, the inundation depths were compared for a variety of rainfall return periods and rainfall ephemerides. Figure 5 illustrates the distribution of the simulated maximum water depth on the ground in the study area. In different rainfall return periods and calendar periods, water accumulated on the ground to a depth of usually less than 0.3 m, followed by 0.3∼0.5 m. The depth of flood inundation increased with the increasing rainfall return period for the same rainfall duration. The depth of flooding increased with the increasing rainfall duration for the same rainfall return period. Overall, the ponded water was primarily concentrated in areas adjacent to rivers and low-lying roads, mainly because the water level of the rivers is higher than the drainage inlets or the terrain of the ponded areas is lower than the surrounding terrain resulting in inundation.

#### 3.1.2. Analysis of the Duration and Extent of Flood Inundation

**Figure 6:**
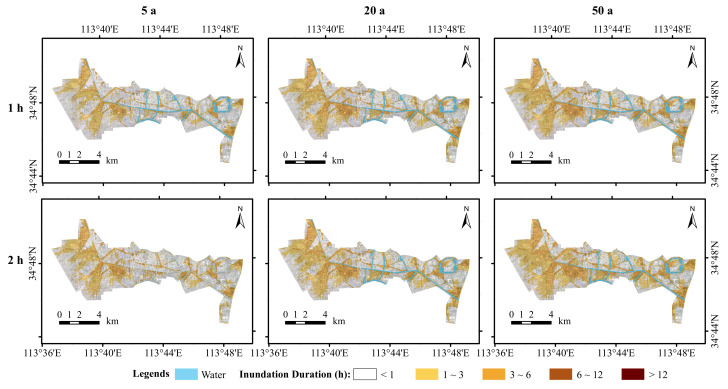
Simulated inundation durations and inundation ranges.

In addition to the inundation depth, the duration of inundation and the inundation extent are essential indicators for evaluating inundation hazards. The amount of precipitation, the type of subsurface, the drainage capacity of the pipe network, and the drainage capacity of natural watercourses are the main causes of persistent flooding. When precipitation is low, the type of subsurface and the drainage capacity of the pipe network control the extent and duration of flooding. Conversely, when rainfall is relatively high, the water level in the river rises and the stormwater pipe network is unable to re-drain or the water in the river can reverse its flow into the stormwater pipe network. At this point, the drainage capacity of the river becomes an important factor in the occurrence and duration of flooding. Analysis of the flood inundation duration and flood extent, therefore, has an important role to play in flood risk assessment. Figure 6 shows the inundation duration and inundation extent of the study area simulated under different scenarios. The simulation results showed that the inundation duration of the study area was mainly from 0∼3 h. At the study area scale, the extent of inundation increased with the duration of rainfall, whereas the extent of flood inundation tended to increase with increasing rainfall return periods. The elevation range of the study area according to the DEM data is 85.05 m 100.34 m. The areas with longer inundation durations are mainly concentrated on the banks of rivers and low-lying areas of the terrain, mainly because the areas with longer inundation durations tend to have deeper inundation, and the areas with deeper inundation generally have lower terrain, which makes it difficult to drain the accumulated rainwater within a short period.

According to the characteristics of precipitation in the study area, normal precipitation can recede within two hours, and precipitation with an inundation time of less than two hours does not affect our normal activities or pose too much of a threat to people’s safety. An inundation time of greater than four hours affects people’s travel activities. In view of the above, we subdivided the inundation time into five ranges [41]. For each inundation calendar time classification, the inundation area was calculated based on the simulation results for the different scenarios of rainfall recurrence. According to Table 3, under the 1 h rainfall calendar, the inundated areas for the rainfall recurrence periods 5 a, 20 a, and 50 a were 4283.11 ha, 4977.61 ha, and 5238.07 ha, respectively, and the total inundation area increased by 954.96 ha from 5 a to 50 a. The inundated areas under the 2 h rainfall duration were 4453.39 ha, 5079.15 ha, and 5527.19 ha, respectively, with a total increase of 1073.8 ha from 5 a to 50 a.

Under the scenarios with rainfall recurrence periods of 5 a, 20 a, and 50 a, the inundated areas with a rainfall duration of 2 h were compared with those with a rainfall duration of 1 h. The simulation results showed that the inundated areas with an inundation duration <1 h decreased by 236.95 ha, 320.04 ha, and 451.65 ha, respectively; the inundated areas with an inundation duration of 1∼2 h increased by 146.41 ha, 110.89 ha, and 170.26 ha, respectively; the inundated areas with an inundation duration of 2∼3 h increased by 100.06 ha, 125.78 ha, and 190.85 ha, respectively; the inundated areas with an inundation duration of 3∼4 h increased by 355.41 ha, 428.08 ha, and 444.54 ha, respectively; and the inundated areas with an inundation duration >4 h decreased by 194.65 ha, 243.17 ha, and 64.88 ha, respectively. The inundated areas with inundation times <1 h and >4 h showed a decrease with increasing rainfall calendar time, indicating that the longer the rainfall calendar time, the faster the drainage of the study area.

The modelling results showed that the inundated areas increased with increasing rainfall return period for the 1–2 h inundation time scenario. For the 1–2 h inundation time scenario, the inundated areas increased by 146.41 ha, 110.89 ha, and 170.26 ha. For inundation durations of 2 to 3 h, the inundated areas increased by 100.06 ha, 125.78 ha, and 190.85 ha. Inundation areas with inundation durations of 3 to 4 h increased by 355.41 hectares, 428.08 hectares, and 444.54 hectares. Inundated areas with inundation durations <1 h and >4 h decreased with increasing rainfall duration, with inundation decreasing by 236.95 ha, 320.04 ha, and 451.65 ha at inundation durations <1 h. The inundated areas with an inundation duration >4 h decreased by 194.65 ha, 243.17 ha, and 64.88 ha. The above results indicate that the longer the rainfall duration, the faster the drainage rate of the study area.

#### 3.1.3. Flood Flow Rate Analysis

**Figure 7:**
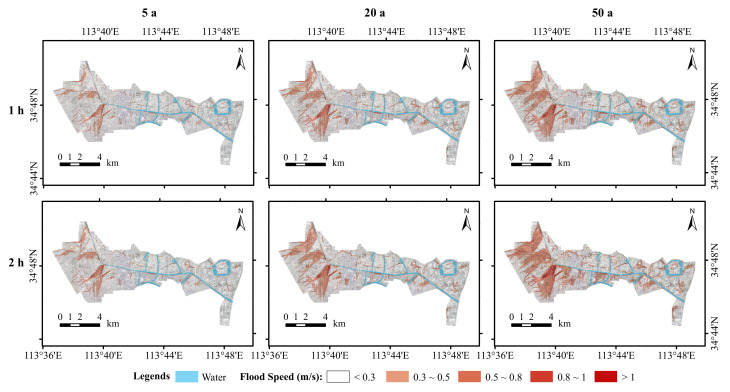
Simulated flood flow map.

Flood flow velocities are an important indicator of urban flood risk conditions [31,42,43]. As shown in Figure 7, the flood flow velocity distributions under different scenarios are shown for the study area. In the study area, the flood flow velocities were usually between 0 and 0.5 m/s for different return periods of rainfall. The flood flow rates tended to increase with increasing rainfall return periods, and increasing rainfall durations also contributed to the increase in the flood flow rates. The areas with a high flood flow velocity are located near the main drainage network’s outlet and in densely populated areas. The magnitude of the flood flow can affect the travel and safety of people in the study area. Excessive flood velocities can impede traffic and float objects on the ground, posing a threat to human life. This is particularly important for the subsequent flood risk analysis.

### 3.2. Analysis of Drainage System Load Conditions

The stormwater pipe network in the study area is mainly located in the paving of main and secondary roads such as the North Third Ring Road, Garden Road, Jing San Road, Zhong Zhou Avenue, Sha Men Road, East Huang He Road, and East Dong Feng Road. Stormwater is discharged into the stormwater pipe network through the catchment area and eventually discharged into the Dongfeng Drain after collection by the stormwater pipe network.

**Figure 8:**
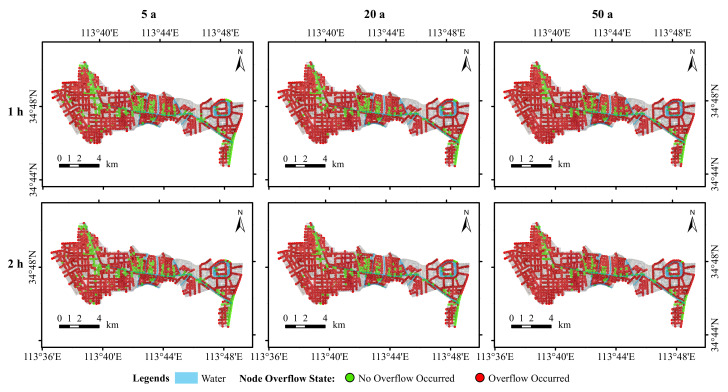
Simulated node overflow condition diagram.

The drainage system load conditions were analysed in terms of both the nodal overflow conditions and drainage network load conditions. Figure 8 presents the distribution of the node overflows in the study area simulated under the different scenarios. It can be seen that most of the nodes showed varying degrees of overflow, which is relatively obvious. According to the statistical results in Table 4, the percentage of overflow at the nodes with a rainfall duration of 1 h increased from 79.01% to 87.36%, whereas the percentage of overflow at the nodes with a rainfall duration of 2 h increased from 79.43% to 87.36% under the conditions of 5 a, 20 a, and 50 a rainfall recurrence periods. Under the same recurrence periods, the proportion of nodal overflows with a 2 h rainfall ephemeris increased by 0.42%, 0.26%, and 1.58%, respectively, compared with those of nodal overflows with a 1h rainfall ephemeris. The overall pattern was that the proportion of nodal overflows increased with the increases in the rainfall return period and rainfall ephemeris.

**Figure 9:**
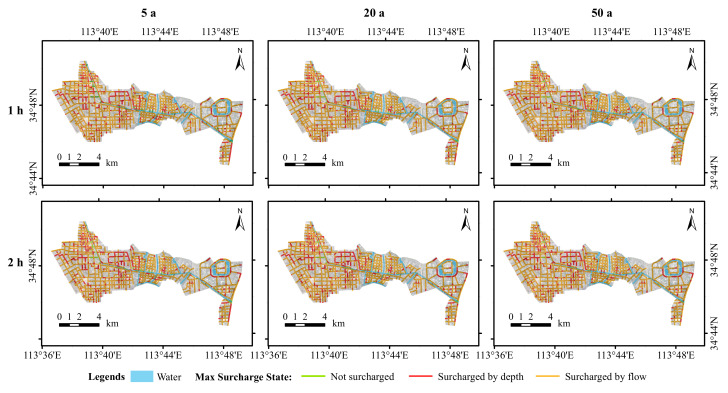
Simulated pipe network load conditions.

In this study, the overload state of the pipe network was judged according to its water flow state. According to the simulation results, when the overload state was less than 1, it meant that the pipe network was in a non-full gravity flow state, which means that the pipe network was in a normal state. When the overload state was equal to 1, the pipe was in a pressure flow state and the hydraulic gradient < pipe slope. The main reason for this state is the insufficient overflow capacity of the downstream pipeline, which causes the pipeline to be in an overload condition. When the overload state was equal to 2, the pipe was in a pressure flow state and the hydraulic slope > pipeline slope; the pipe was overloaded due to its own drainage capacity being insufficient. The pipe network was generally considered to be overloaded in the case of overload states 1 and 2. As can be seen in Figure 9, the pipe networks in the study area were generally in an overload condition, with the flow overload being the dominant overload pipe network. The length of the overloaded pipe network increased with increasing precipitation return periods.

According to the overload statistics of the pipe network under rainfall return periods of 5 a, 20 a, and 50 a, as seen in Table 5, the overload water depth lengths of the pipe network with a rainfall duration of 1h were 49.07 km, 42.38 km, and 38.81 km, and the corresponding overload flow lengths of the pipe network were 241.33 km, 249.56 km, and 253.87 km. The overload flow lengths of the pipe network corresponding to 2 h were 235.16 km, 239.91 km, and 245.25 km. The above results show that the length of the overloaded pipe network increased with the increase in the rainfall return time, whereas the length of the overloaded pipe network decreased with the increase in the rainfall recurrence period.

### 3.3. Urban Flood Risk Analysis

The flood risk rate analysis method proposed by the UK EPA has been used to express the flood risk rate as a combination of two key physical quantities, namely water depth and flow velocity. The type of subsurface is also considered to be an important factor influencing the flood risk rate. Finally, an RH value characterising the flood risk was calculated to characterise the degree of flood risk.

**Figure 10:**
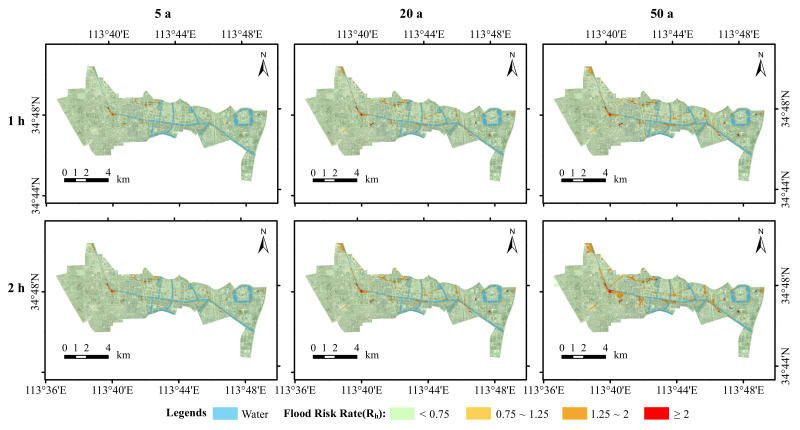
Inundation flood risk for different scenarios in the study area.

Figure 10 shows the distribution of the flood risk in the study area, simulated under the different scenarios. It can be seen that the distribution of risk is dominated by low-risk areas with very few very high-risk areas. The medium- and high-risk areas are mainly located on the banks of rivers, in built-up areas, and on low-lying urban terrain.

A detailed analysis of the change in the flood risk area during the rainfall return period for the 5 a, 20 a, and 50 a scenarios is presented based on the simulation results in Table 6. For a rainfall duration of 1 h, the low-risk areas were 7195.39 ha, 7058.04 ha, and 6959.16 ha and the medium-risk areas were 51.44 ha, 87.18 ha, and 101.74 ha, respectively. At a rainfall time of 2 h, the low-risk areas were 7148.54 ha, 6981.60 ha, and 6740.90 ha; the medium-risk areas were 62.38 ha, 107.33 ha, and 125.89 ha; the high-risk areas were 112.65 ha, 225.05 ha, and 430.37 ha; and the very-high-risk areas were 14.75 ha, 24.33 ha, and 41.16 ha, respectively.

The shift in the rainfall return periods from the 5 a to the 50 a risk zones was then analysed under the same rainfall calendar. For a rainfall duration of 1 h, the shifts from low- to medium-, high-, and very-high-risk zones were 99.19 ha, 134.43 ha, and 2.61 ha, respectively; from medium- to high- and very-high-risk zones were 48.08 ha and 0.80 ha, respectively; and from high- to very-high-risk zones was 13.01 ha. With a rainfall duration of 2 h, the shifts from the low-risk zone to the medium-, high-, and very-high-risk zones were 124.84 ha, 277.79 ha, and 5.01 ha, respectively; from the medium-risk zone to the high- and very-high-risk zones were 60.30 ha and 1.02 ha, respectively; and from the high-risk zone to the very-high-risk zone was 20.38 ha. The combination of the risk distribution ranges in Figure 10 indicates that the upgraded risk areas are mainly located on both sides of the river and in the study area, which are low-lying and have poor drainage capacity.

There are also some characteristics of the change in the flood risk zones from a 1 h to a 2 h rainfall ephemeris for the same rainfall return period. When the rainfall return period was 5 a, 34.58 ha and 12.32 ha of low-risk zone converted to medium- and high-risk zones, respectively, and 0.05 ha of medium-risk zone converted to low-risk zone, 23.45 ha to high-risk zone, and 0.14 ha to very-high-risk zone. When the rainfall return period was 20 a, the areas of low-risk zone converted to medium- and high-risk zones were 58.31 ha and 18.02 ha, respectively, and the area of medium-risk zone converted to high-risk zone was 38.39 ha. When the rainfall return period was 50 a, the areas transformed from low-risk areas to medium- and high-risk areas were 104.15 ha and 113.66 ha, respectively, and the area transformed from medium-risk area to high-risk area was 79.99 ha. The area transformed from medium-risk zone to high-risk zone was 79.99 hectares. Combined with Figure 10, it appears that the risk areas close to the river are more likely to be upgraded, and the high-risk areas are also generally concentrated near the river.

The above results show that for the same rainfall return period, the area covered by low-risk areas decreased with increasing rainfall calendar time, and most of the reduced low-risk areas transformed into medium- and high-risk areas. The area covered by all the risk classes, except for low-risk areas, increased with increasing rainfall calendar hours. For the same rainfall calendar time, the area covered by low-risk areas decreased with increasing rainfall return periods. The area covered by all risk classes, except for low-risk areas, increased with increasing rainfall return periods. The above analysis indicates that increases in the rainfall return period and rainfall duration will result in more severe flooding. It is also important to focus on high- and very-high-risk areas when undertaking flood management and when flooding occurs. It is important to consider that the flood risk varies with the duration and intensity of rainfall ephemeris. It is therefore also important to focus on flooding in low-risk areas that could easily convert into high-risk areas.

## 4. Conclusions

In this study, a 1D/2D coupled urban flood model was constructed using InfoWorks ICM software based on data pertaining to pipe networks, inspection wells, roads, water systems, land uses, and elevations in the Dongfeng Canal area of Zhengzhou. Six scenarios were set up according to different rainfall recurrence periods and rainfall ephemeris using the Zhengzhou storm intensity formula. According to the simulation results, the flood inundation depth, inundation extent, duration of inundation, flood flow, and drainage system load were analysed. Finally, the flood risk was quantified and spatially analysed using the flood risk rate analysis method proposed by the UK EPA. The main findings of this study are as follows.

(1) This study uses the InfoWorks ICM model to construct a coupled hydrological-hydraulic model for a small urban watershed area. The two-dimensional hydraulic processes of the urban one-dimensional pipe network, river, and surface are coupled and the model effects are compared using the flood inundation locations of historical precipitation. The results show that the model is effective in simulating rainfall and flooding in the Dongfeng Canal area. The model can be applied to the analysis of flood risk.

(2) According to the simulation results, inundation depths in the study area are mainly 0∼0.3 m, followed by 0.3∼0.5 m. Inundation is mainly concentrated in areas near rivers and low-lying areas of road topography. The extent of inundation in the study area at a 2 h rainfall ephemeris is greater than that at a 1 h rainfall ephemeris, and the extent of inundation tends to increase with increasing recurrence periods. The extent of inundation at <1 h and >4 h appears to decrease with increasing rainfall ephemeris, indicating that the longer the rainfall ephemeris, the faster the study area drains. The distribution of flood velocities shows a tendency to increase with increasing return periods, with higher velocities being distributed mainly in drainage inlets near the main drainage network and densely built-up areas.

(3) The proportion of nodal overflows occurring increases with increasing rainfall return periods and rainfall ephemeris. The pipe network in the study area is generally overloaded under the different scenarios, with flow overloading dominating and the length of the overloaded pipe network increasing with the rainfall return period. The length of the bathymetric overload pipe network increases with increasing rainfall return periods, whereas the length of the flow overload pipe network decreases with increasing rainfall return periods.

(4) The distribution of the flood risk in the study area is dominated by low-risk areas, with very few very high-risk areas. The medium- and high-risk zones are mainly located on both sides of the river in built-up areas and low-lying urban areas. The simulation results under the different scenarios show that the areas of medium-, high-, and very-high-risk zones increase with the increasing rainfall return period and rainfall duration. The area of low-risk zones decreases with the increasing rainfall return period and rainfall duration, and most of the reduced low-risk zones are transformed into medium- and high-risk zones. Increases in rainfall return periods and rainfall durations can lead to more severe flooding. It is therefore important to focus on high- and very-high-risk areas, as well as low-risk areas that can easily transform into high-risk areas when managing floods when flooding occurs.

This study analyses the flood risk situation in the study area in various aspects and from various perspectives. It can provide a reference for the prevention and control of flooding and the formulation of flood countermeasures in the area and help to improve the management of flood risks in the urban construction process. At the same time, the model lacks further validation due to limited measurement data. The construction of the flood risk indicators is relatively singular and does not take into account more comprehensive factors such as socio-economic factors. Therefore, a more detailed collection of hydrological and socio-economic data in the study area to further improve the accuracy of the model and construct a more comprehensive urban flood risk system will be the focus of our work in the next phase.

## Figures and Tables

**Table 1:** Model production sink parameters.

Type of Landuse	Type of Maternal Flow	Production Flow Parameters				Runoff Routing Value
		Fixed Runoff Coefficient	Initial Infiltration Rate	Stable Penetration Rate	Attenuation Factor	
Building site	Fixed	0.9	-	-	-	0.019
Road		0.9	-	-	-	0.02
Green land	Horton	-	76.5	2.5	2	0.13
Bare area		-	65	2.5	2	0.05

**Table 2:** Flood risk classification.

Inundation Hazard Index Threshold ($R_{h}$)	Risk Level	Description
<0.75	Low risk	Shallow standing water or the presence of shallow static waterlogging
0.75∼1.25	Medium risk	Deep water or fast-flowing water
1.25∼2.0	High risk	Hazardous area with deep water and high flow rates
≥2.0	Very high risk	Very dangerous area, no access

**Table 3:** Flooded area statistics for different scenarios (ha).

Rainfall Return Period	Duration of Rainfall	Duration of Inundation					Area Inundated
		<1	1∼2	2∼3	3∼4	>4	
5a	60	1734.56	983.55	500.85	431.26	632.89	4283.11
	120	1497.61	1129.96	600.91	786.67	438.24	4453.39
20a	60	1736.95	1264.00	558.58	444.71	973.37	4977.61
	120	1416.91	1374.89	684.36	872.79	730.20	5079.15
50a	60	1696.68	1368.74	590.29	410.83	1171.53	5238.07
	120	1245.03	1539.00	781.14	855.37	1106.65	5527.19

**Table 4:** Overflow statistics for different scenario nodes.

Rainfall Return Period	Duration of Rainfall (min)	Number of Overflows Occurring at Nodes	Proportion of Overflows Occurring at Nodes
5a	60	1506	79.01%
	120	1514	79.43%
20a	60	1593	83.58%
	120	1598	83.84%
50a	60	1635	85.78%
	120	1665	87.36%

**Table 5:** Statistics for overloading of the pipe network for different scenarios.

Rainfall Return Period	Duration of Rainfall (min)	Length of Pipe Network Overloaded by Water Depth (km)	Length of Pipe Network with Flow Overload (km)	Total Length of Overloaded Pipe Network (km)
5a	60	49.07	241.33	290.4
	120	55.44	235.16	290.6
20a	60	42.38	249.56	291.94
	120	52.43	239.91	292.34
50a	60	38.81	253.87	292.68
	120	48.17	245.25	293.42

**Table 6:** Area statistics for inundation flood risk rates for different scenarios (ha).

Rainfall Return Period	Duration of Rainfall (min)	Flood Risk			
		Low Risk	Medium Risk	High Risk	Very High Risk
5 a	60	7195.39	51.44	81.96	9.53
	120	7148.54	62.38	112.65	14.75
20 a	60	7058.04	87.18	173.29	19.81
	120	6981.60	107.33	225.05	24.33
50 a	60	6959.16	101.74	251.47	25.95
	120	6740.90	125.89	430.37	41.16

## Data Availability

Not applicable.
